# Structured surgical residency training in Germany: an overview of existing training programs in 10 surgical subspecialties

**DOI:** 10.1515/iss-2018-0033

**Published:** 2019-05-10

**Authors:** Sabine Drossard

**Affiliations:** Klinik für Kinderchirurgie und Kinderurologie, Universitätsklinikum Augsburg, Stenglinstraße 2, 86156 Augsburg, Germany

**Keywords:** competency-based education, continuing medical education, patient safety, postgraduate medical education, specialty training, structured residency program, surgical education

## Abstract

**Introduction:**

Surgery and the training of young surgeons face various challenges. Work hour restrictions, demographic changes, medicolegal demands, as well as economic constraints have led to changes in patient care and thus changed surgical residency. In addition to the daily training at work, several theoretical and practical courses are offered to surgical residents. The aim of this manuscript is to provide an overview of the existing courses and programs for surgical residents in Germany. It describes the current structure of surgical training in Germany for 10 subspecialties and sets out existing approaches to implement structured surgical training by professional associations as well as by commercial providers.

**Materials and methods:**

The official homepages of 10 surgical associations were analyzed for information on structured surgical training and a Google search for surgical training programs was conducted. Then, the websites of two commercial providers were searched for information in courses. In addition, the members of the German Young Surgeons Association were asked open questions about the existence of structured resident training and additional training opportunities in their specialty. The courses were analyzed for structural characteristics such as the price and type of course (single course, exam preparatory course, and structured program). A structured program was defined as a set of courses based on learning objectives that are designed to cover all aspects of the specialty and include some form of summative or formative assessment.

**Results:**

Several courses are offered by varied providers; some of them associated with surgical associations and some commercial. Seven of 10 professional associations offer single courses and six of them offer exam preparatory courses. Commercial providers only offer single courses. All of these courses are optional; there is no requirement to take part in any of them. None of them is free of charge, but most offer discounts for members of surgical societies. Only one structured program exists for orthopedics and trauma surgery. A fixed schedule does not exist for any surgical subspecialty, but it is rather the responsibility of the trainee or his/her supervisor to pick or suggest a course that suits their personal state of knowledge.

**Discussion:**

Until now, it depends on personal motivation and the generosity of hospitals whether or not surgical residents will receive training outside of their training hospital. Various external courses are offered in all surgical subspecialties to complement on-the-job training. It is unknown how many residents take part in them. The implementation of new, competence-based specialist training regulations in Germany in 2018 may facilitate a change in surgical education. Simulation-based education can promote the acquisition and consolidation of surgical skills. Additional training possibilities and structured programs should be implemented in surgical resident training to foster competence-based education and surgical proficiency.

## Introduction

Surgical training has to equip trainees with the skills, knowledge, and competencies necessary to provide high-quality patient care.

During training, a surgical resident will have to not only develop technical skills and acquire factual knowledge but also develop the ability to make decisions on who needs to be operated on and to select the appropriate procedure. They have to be able to handle routine as well as complicated postoperative care and surgical emergencies.

Surgery has had a long established “apprenticeship model” of training: the trainee would learn through observing and imitating the work of a skilled mentor [1]. That means surgical trainees gained knowledge and most of their skills from older surgeons’ expertise and by being immersed in the clinical environment for prolonged periods of time. With patient safety in mind and regarding working time regulations and shift work and increasing procedural complexity, this approach is no longer feasible nor educationally acceptable [2].

The “new” European working time directive, which took effect in 2003, restricts work hours to 48 h per week including on-call shifts [3]. Furthermore, surgical trainees on rotating shifts are often not able to attend scheduled learning opportunities such as grand rounds, radiology meetings, mortality and morbidity conferences, lectures, and tutorials. Thus, on-the-job training opportunities have diminished, while at the same time surgical care has become more complex because of growing knowledge and the implementation of new methods: “Loss of week day training and decrease in overall length of training due to restricted hours has occurred when the complexity of the knowledge base and skill levels for surgical training has historically increased” [4]. In a nationwide survey among residents by the German Medical Association (Bundesärztekammer, BÄK) in 2011, 66% of residents stated that they feel they are not able to accomplish their postgraduate education in their appointed work hours [5].

In the last decade, several countries have changed their surgical training and implemented competency-based surgical training models, usually with hands-on training and operative skills assessment in surgical skills labs. Surgical training in the United Kingdom, for example, has seen profound changes after the introduction of formal training curricula and competency-based assessments [6]. In Ireland, surgeons are expected to complete lab-based operative skills assessments and “Competence Assessment and Performance Appraisals”, which involve the so-called “Supervised Structured Assessments of Operative Performance” [7].

In Germany, the reorientation of specialty training from the current checklist-like model to competency-based curricula is currently taking place. After long and intensive discussions, new specialty training regulations (Weiterbildungsordnung, WBO) have been proposed by BÄK in 2018. Levels of competencies have been defined as essential outcomes of postgraduate training. The regulations focus on educational goals rather than on the duration of training; numerical values for medical procedures have been reduced [8]. In a next step, the regional state medical chambers will have to implement the new regulations and put them into practice.

### Surgical training in Germany

Postgraduate training in Germany, unlike in other countries, is not associated with universities. It focuses almost entirely on work-based learning without formal taught courses.

The content, duration, and objectives of specialty training are proposed by the BÄK and specified in their (model) WBO [(Muster-) WBO]. The 17 regional state medical chambers (Landesärztekammern) are responsible for the implementation of the definitive WBO for every state. There are only small differences in the training regulations between regions.

Surgical education is recognized only by authorized surgeons (Weiterbildungsermächtigte). A specific number of procedures to be completed by the end of surgical training is specified in the training regulations (WBO). The required time, knowledge, and skills are documented in a logbook (“catalogue”) that allows surgical trainees to record their operative experience by documenting the procedures they have performed. The completion of the catalogue is required to apply for the exam to become a certified specialist. A final oral exam is held by a three-person board selected by the medical chamber of the region (Bundesland) where the trainee worked at the end of their training.

In Germany, there are 10 surgical subspecialties: general surgery, visceral surgery, cardiac surgery, thoracic surgery, pediatric surgery, orthopedics and trauma surgery, plastic surgery, vascular surgery, neurosurgery, and oral and maxillofacial surgery.

Postgraduate surgical training of the first eight subspecialties is organized concertedly, whereas neurosurgery as well as oral and maxillofacial surgery has separate training structures.

The concerted surgical training in Germany takes 6 years, and most of the training is completed in hospitals. The first 2 years consist of basic clinical training, the so-called “common trunk”, which encompasses 6 months in an emergency department, 6 months in an intensive care unit (ICU), and 1 year in a surgical ward. This part of the training can be carried out in any department of the eight mentioned subspecialties. Nevertheless, most residents will complete their “common trunk” in the specialty they have chosen. After the common trunk, residents can select a specialty for further training. The following 4 years are structured differently according to specialty, but the main part will be carried out in a surgical department of the chosen specialty. Training in neurosurgery also takes 6 years, including 6 months of training in the ICU. To some extent, external rotations are acceptable (e.g. up to 12 months of training in neurosurgery for orthopedics and trauma surgery) or even mandatory (e.g. 12 months of training in pediatrics for pediatric surgeons) [9].

During their training, the trainees are employed at a hospital where they usually work full-time in patient care (outpatient clinic, emergency room, ward, operation theater, and on-call shifts). Much of the learning is implicit: residents observe more senior doctors treating patients or receive feedback on their own activities. They then gradually deduce appropriate treatment plans and actions for any given case from their observations and experiences.

### Challenges

The working environment of the German health-care system threatens high-quality surgical training and education: the implementation of Diagnosis-Related Groups in 2004 has led to shorter hospital stays, whereas the number of admissions has risen [10]. That means more patients are admitted and discharged every day and only the sickest patients, many of them requiring complex care, remain in hospital. The reduction in working hours by working time regulations and the increasing workload put on hospital doctors have led to challenges, particularly in surgical training.

The Professional Association of German Surgeons (Berufsverband der Deutschen Chirurgen e.V., BDC) conducted a survey among surgical trainees and young surgeons who have recently been board approved in Germany in 2014/2015. Only 30% of the 1100 participants felt that their training hospital cares about the length of their training. Sixty percent of the respondents felt that the “new” working time directive has decreased their time spent in the operating room (OR). Furthermore, two thirds (67%) of young surgeons stated that no general structure for their training exists in their teaching hospital. Eighty-six percent of the respondents were not provided with a training curriculum at the beginning of their training (which is mandatory). There have been practically no changes regarding the aspect of training structure in comparison to precedent surveys [11].

### Aim

This manuscript describes the current structure of surgical training in Germany and sets out different approaches to implement structured postgraduate training. It is based on information that is freely available online as well as personal experience and solicited statements by young surgeons in training, but the author did not receive information from trainees of all subspecialties. It provides general information about surgical training in Germany and makes no claim to be complete.

The aim of this manuscript is to provide an overview of existing courses and programs for surgical residents in Germany. The current structure of training offered by professional associations as well as by some commercial providers is described.

## Materials and methods

An online research was carried out. The official homepages of the 10 surgical associations as well as the website of the German Society for Surgery (Deutsche Gesellschaft für Chirurgie) were analyzed for information on structured surgical training. Furthermore, a Google search for surgical training programs was conducted in German using the words “Strukturierte+Weiterbildung+Chirurgie”, “Weiterbildungsangebot+Chirurgie”, and “Akademie+Chirurgie” and separately for every subspecialty (“Weiterbildung Kinderchirurgie”, “Weiterbildung Unfallchirurgie”, etc.).

In addition, the members of the German Young Surgeons Association (Perspektivforum Junge Chirurgie, PFJC) were asked about the existence of structured resident training in their specialty. They were asked open questions (“In what form, if any, does structured resident training exist in your specialty? Please specify on its form and whether you have participated in it. How do you rate the implementation of the structured programs?”).

This information was used to confirm and complement the collected data from the WebSearch. The websites of commercial programs that were named were analyzed as well.

The PFJC is an association of young representatives of the surgical societies. At the time of research, there were 24 members of 10 surgical subspecialties. Personal information was obtained for vascular surgery, orthopedics and trauma surgery, general and visceral surgery, plastic surgery, oral and maxillofacial surgery, and pediatric surgery. In addition, there was personal insight on the training programs of the BDC. The representatives of the other surgical subspecialties did not respond to the request.

The results are presented in Table 1. A structured program was defined as a set of courses based on learning objectives that are designed to cover all aspects of the specialty and include some form of summative or formative assessment.

**Table 1:** Overview of surgical training courses in Germany prices according to the websites of the societies as of 2018.

	Single courses	Structured program	Exam preparatory courses	Mandatory	Free of charge	Typical price (full/discounted)	Discount for associated members
Professional associations							
BDC (various)	✔	-	✔	-	-	500/300–650/500 €	✔
DGG (vascular surgery)	✔	-	-	-	-	395–750/495 €	✔
DGKCH (pediatric surgery)	-	-	-	-	-	140/70 €	✔
DGAV (general and visceral surgery)	✔	-	✔	-	-	425/350–900/800 €	✔
DGMKG (oral and maxillofacial surgery)	✔	-	✔	-	-	550/330 €	✔
DGNC (neurosurgery)	-	-	-	-	-	350 €	-
DGOU (orthopedics and trauma surgery)	✔	✔	✔	-	-	550 €	-
DGPRÄC (plastic surgery)	✔	-	✔	-	-	25 €	-
DGT (thoracic surgery)	-	-	-	-	-	–	–
DGTHG (cardiac surgery) in association with Aesculap Academy	✔	-	✔	-	-	290/250–650/500 €	✔
Commercial							
VI (vascular surgery)	✔	-	-	-	-	500–1550 €	-
Aesculap Academy (various)	✔	-	-	-	-	100/80–1100/1020 €	✔^a^

^a^The Aesculap Academy offers discounts for the members of the DGG, DGTHG, DGOU, DGAV, CAMIC, and more depending on the courses offered.

## Results

There are different approaches to implement structured surgical training in Germany. Several courses are offered by varied providers; some of them associated with surgical associations and some commercial. Table 1 gives an overview of the available courses in Germany.

All of these courses are optional, and there is no requirement to take part in any of them. A fixed schedule does not exist for any surgical subspecialty. It is rather the responsibility of the trainee or his/her supervisor to pick or suggest a course that suits the personal state of knowledge. None of the courses are free of charge.

The German Society for Orthopaedics and Trauma Surgery (Deutsche Gesellschaft für Orthopädie und Unfallchirurgie, DGOU) is the only professional association to offer a structured program that is based on clearly defined learning objectives, contains both theoretical and practical aspects, and includes exams.

### BDC

The German BDC offers a wide array of surgical courses as well as leadership and management trainings.

Their so-called “Academy for Continuing Education in Surgery” (BDC|Akademie) offers courses for students and residents as well as experienced surgeons [12].

A broad range of practical courses from basic techniques to highly specialized courses from all walks of surgery (i.e. minimally invasive techniques, osteosynthesis courses, surgical intensive care, and many more) complement on-site surgical training. For example, the BDC offers a range of laparoscopic training courses certified by the Surgical Working Group for Minimally Invasive Surgery (Chirurgische Arbeitsgemeinschaft für Minimal-Invasive Chirurgie, CAMIC) of the German Society for General and Visceral Surgery (Deutsche Gesellschaft für Allgemein- und Viszeralchirurgie, DGAV).

Apart from practical teaching, the BDC|Akademie also offers a range of preparatory tutorials for board examinations in seven of eight surgical specialties [in pediatric surgery, the German Society of Pediatric Surgery (Deutsche Gesellschaft für Kinderchirurgie, DGKCH) offers their own course; see below].

BDC|Akademie courses are popular. In 2017, almost 3000 surgeons participated in 194 seminars [13].

#### Vascular surgery

For residents in vascular surgery organizations such as Vascular International (VI), the German Society of Vascular Surgery & Medicine (DGG), the Professional Association of German Surgeons (BDC), and the German Society of Ultrasound in Medicine (Deutsche Gesellschaft für Ultraschall in der Medizin, DEGUM) offer competing and additional courses.

VI is a private academy that offers bilingual (English and German) hands-on trainings mostly in Germany, Switzerland, and Austria. The most popular courses are the basic and the masterclass. The basic class provides basic techniques for vascular surgery and is suitable for trainees in the first 3 years. The masterclass teaches the essential surgical procedures of the specialty and is recommended in the final phase of training {Anonymous:oWzeTxGm}.

The DEGUM provides several ultrasound courses specially designed for vascular surgeons matching different stages of training.

The DGG provides hands-on courses, ultrasound courses, and workshops for residents; some of them are in cooperation with the above-mentioned societies {Anonymous:DyNX_i-m}.

The European Vascular Course offered by the European Society for Vascular Surgery offers both lectures and hands-on courses. There are teaching sessions covering all aspects of the field and case discussion rounds in an international setting [14].

Table 2 gives a brief overview of the educational possibilities. It includes the recommended state of training in which a certain course should be considered.

**Table 2:** Timeline for training in vascular surgery.

**Training**						**Exam**	**Vascular surgeon**
**1st year**	**2nd year**	**3rd year**	**4th year**	**5th year**	**6th year**		
					
Common trunk		Special training (vascular surgery)					Further specialization
						
					Preparation for final exam (DGG/BDC)		Fellow of the European Board of Vascular Surgery
Ultrasound course (DEGUM/DGG)						
Basic class (VI)				Masterclass (VI)			Courses for specialists (DGG/VI)
	Vascular access, open/endovascular surgery (VI/DGG)						
			European vascular course				European vascular course
≫Timeline≫							

#### Pediatric surgery

The DGKCH offers a structured training program for residents in pediatric surgery.

The Academy for Pediatric Surgery (Akademie für Kinderchirurgie, AKIC) is a 2-day course specifically designed for residents and held once per year. Experts in pediatric surgery (mainly department heads of pediatric surgery in Germany, Austria, and Switzerland) will give lectures and provide workshops to prepare residents for their final exams. The subjects are chosen in a way that if a resident participated in this course four to five times he or she should have attended lectures about almost all major subjects of pediatric surgery.

In 2014, the AKIC was given a clear structure. Four different categories were defined:

1. Head, neck, and thorax;
2. Urology and oncology;
3. Abdomen and gastrointestinal tract; and
4. Traumatology and musculoskeletal disorders.

The course was established in 1966. In 2019, the 54th AKIC will take place. Every year, more than 150 residents attend. The course takes 2 days. Participation costs 140 € (70 € for members of the DGKCH) [15].

The program focuses mainly on knowledge. Because of the high number of participants, divergent skills levels, and limited time, the AKIC provides only little skills training. Lectures are designed to present consolidated knowledge, whereas workshops give the opportunity for more in-depth discussions on selected aspects of pediatric surgery. There is no exam.

#### General and visceral surgery

The DGAV provides a variety of courses for residents. They are structured in the Center for Continuing Medical Education and Quality (Weiterbildungs-, Fortbildungs- und Qualitätszentrum). The training concept for residents is divided in theory, technique, anatomy, practical workshops, and preparation for the board certification.

This includes a weeklong course named “practical course for visceral surgery” where residents are trained in conventional and laparoscopic techniques of visceral surgery, including basic skills of thoracic and vascular surgery. In addition to the practical courses, there are a variety of lectures on state-of-the-art concepts and operative strategies.

A modular learning concept that uses modern didactic methods for online-based as well as classroom-based seminars exists for theoretical courses. There are different technique courses for each surgical field (e.g. upper gastrointestinal), all of them focus on teaching practical skills on animal models. The anatomy courses are held by an anatomist together with a surgical expert. Participants get to refresh their knowledge on topography of a certain region on cadavers before moving on to patients. The practical workshops focus on the improvement of specific operative skills and are offered for different levels of training: for beginners (during residency), for adepts (before or immediately after board certification), and for experts (specialists). Additionally, the DGAV offers courses to prepare for the board certification with a comprised overview of the relevant knowledge and interactive case presentations [16], [17].

#### Orthopedics and trauma surgery

Postgraduate training in orthopedics and trauma surgery in Germany was adapted and reorganized to match similar residency programs in the European Union in 2003 [18]. To provide young surgeons with more flexibility to work in different surgical specialties and to allow for easier migration within the European Union, a common curriculum was created and implemented by all German State Chambers of Medicine in 2006.

The DGOU offers residents a training series called “Fit After Eight”. It was initiated in 2008 and offers young surgeons specializing in orthopedics and trauma surgery structured training parallel to their hospital resident training. The program aims to consolidate and enhance the skills and knowledge required in the final exam and their daily work as specialists. The course consists of eight modules and is based on a catalogue of learning objectives validated by experts. It is offered in four different 4-day courses (each course contains two modules) and takes place in different hospitals in Germany. There are two courses every year so completing all modules takes 2–4 years. The course aims to be problem oriented and in step with actual practice. Residents learn in small groups of 8–10 participants. Each module ends with a combined written and oral exam. The course is endorsed by the German Society for Orthopaedics and Orthopedic Surgery (Deutsche Gesellschaft für Orthopädie und orthopädische Chirurgie), the German Trauma Society (Deutsche Gesellschaft für Unfallchirurgie), and the Professional Association for Orthopaedics and Trauma Surgery (Berufsverband der Orthopäden und Unfallchirurgen, BVOU) [19], [20].

#### Plastic surgery

The German Association of Plastic, Reconstructive and Aesthetic Surgeons (Deutsche Gesellschaft der Plastischen, Rekonstruktiven und Ästhetischen Chirurgen, DGPRÄC) offers nearly 100 courses per year in all subspecialties, i.e. reconstructive surgery, aesthetic surgery, hand surgery, and burn surgery. The course program is sent to all associate members once a year. These courses are organized by different plastic surgery centers in Germany as well some hospitals outside of Germany. The courses are for associated members only and held in small groups of maximum eight people; the fee is 25 € per course. Attending courses of all four subspecialties is encouraged.

As not every hospital that trains plastic surgeons offers all aspects of plastic surgery, the DGPRÄC encourages residents to work and train at different hospitals during their residency with an exchange program (“Rotationsbörse”).

A preparation course for the board exam is held once a year since 2018 [21].

#### Thoracic surgery

The German Society for Thoracic Surgery (Deutsche Gesellschaft für Thoraxchirurgie, DGT) has founded the Academy for Thoracic Surgery (Akademie für Thoraxchirurgie) in 2014 and aims to implement courses for thoracic surgeons as well as certifying courses for thoracic surgeons offered by other providers to ensure their quality and suitability [22].

#### Cardiac surgery

The German Society for Thoracic, Cardiac and Vascular Surgery (Deutsche Gesellschaft für Thorax-, Herz- und Gefäßchirurgie, DGTHG) does not offer any resident courses at the moment [23]. There is, however, a structured program consisting of several courses offered by the commercial provider “Aesculap Academy” that have been developed in cooperation with DGTHG. There are several courses (basic, refresher, and special) and an exam preparatory course consisting of two parts: theory and practice. Each part takes 3 days. Members of DGTHG are offered discounts for these trainings [23], [24].

#### Neurosurgery

Specialist training in neurosurgery is not part of the “common trunk” structure but similar to those surgical specialties. It takes a minimum of 6 years; up to 12 months of training in other specialties are eligible.

The Academy for Neurosurgery (Neurochirurgische Akademie) is sustained by the German Society for Neurosurgery (Deutsche Gesellschaft für Neurochirurgie, DGNC) as well as the Professional Association of German Neurosurgeons (Berufsverband Deutscher Neurochirurgen). Once per year, it offers a training course oriented toward residents in training. A series of five courses cover essential aspects in all areas of neurosurgery, and lectures are held by relevant specialists. The 3-day program usually covers one large and one to two smaller areas of neurosurgery; the course fee is 350 € [25].

#### Oral and maxillofacial surgery

In Germany, one has to complete two degrees, human and dental medicine, to specialize in maxillofacial surgery. Postgraduate training takes 5 years. Therefore, training to become a maxillofacial surgeon lasts between 11.5 and 17 years depending on the order of qualifications (see Table 3). While studying dental medicine, trainees usually work part-time, which shortens the training period; this is not possible if dental medicine was studied first.

**Table 3:** Timeline for studying and training in oral and maxillofacial surgery.

Option 1	Human medicine	Dental medicine	Postgraduate training	Total time
Years	6.5	5.5 (2.5)	5 (2.5)	11.5
**Option 2**	**Dental medicine**	**Human medicine**	**Postgraduate training**	**Total time**

Years	5.5	6.5	5	17

The German Society for Oral and Maxillofacial Surgery (Deutsche Gesellschaft für Mund-, Kiefer- und Gesichtschirurgie, DGMKG) holds annual conferences as well as training courses for subspecialties (cancer treatment, reconstructive surgery, traumatology, implantation, and special radiology courses). Cooperative events are held with other national and international organizations.

Twice a year, synoptic courses prepare for the final exam with lectures on all major subjects of oral and maxillofacial surgery (“MKG Update” and “MKG Kompakt”). Members of the Young Maxillofacial Surgeons Association within the DGMKG try to harmonize training contents and offers (e.g. with a checklist for on-call duties and special meetings for young surgeons within established conferences).

### Commercial providers

There are a number of commercial offers for resident training courses. Some focus on one specialty (e.g. VI), whereas others focus on certain aspects of surgery, e.g. laparoscopic and endoscopic surgery or endoscopy (e.g. courses offered by manufacturers such as B.Braun, Johnson&Johnson, Olympus, and others).

The broadest variety of surgical courses in Germany is offered by the commercial provider “Aesculap Academy” by B.Braun. Courses include basic and advanced laparoscopy courses and special trainings such as advanced laparoscopy for pediatric surgeons or endoprosthetic courses for orthopedic surgeons. As mentioned above, different trainings, courses, and revision courses for cardiac surgery residents are offered in cooperation with the DGTHG [24].

### Train-the-trainer programs

To improve surgical training, the medical societies recommend “Train-the-Trainer” programs. However, there are not many programs available.

The BDC initiated together with the Professional Association of German Internists (Berufsverband Deutscher Internisten) and the BVOU a train-the-trainer concept, called “Mastertrainer”, which is aimed at teaching concepts for a better structured resident training to chiefs and attendings (i.e. the doctors responsible for residency training) [26].

## Discussion

This manuscript describes the current structure of postgraduate surgical training in Germany and sets out different approaches to implement structured surgical education.

It does not claim to be complete; the data are limited to information available on the Internet, literature, and personal statements. It is intended to act as a first comprehensive overview of available surgical training programs for different subspecialties in Germany. However, it can only be perceived as a basis for further research.

The current structure of postgraduate medical education in Germany does not require any external courses or training programs in addition to on-the-job training. Until now, no competency-based assessments exist.

Most of the professional associations and surgical societies have implemented some kind of structured surgical training or at least offer theoretical and practical courses for residents and certified surgeons. But until now, all of these courses are optional and most of them are paid-for. That is to say, it depends on residents’ motivation and on the generosity of hospitals (absence, course expenses) whether or not surgical residents will receive training outside of their training hospital.

Nonetheless, many residents in surgical training attend external training courses. According to a survey that was carried out by BDC in 2014/2015, 95% of surgical residents in Germany state that their employer offers them paid leave to attend external trainings, whereas 82% are granted (partial) financial support [11]. How many residents ultimately take part in structured programs or are able to attend external training courses is not known; currently, there are no data available.

The quantity and variety of courses offered vary considerably between different specialties, as does their pricing policy. Course fees vary between 25 and 1550 € depending on the length, complexity, and expense.

Information about training opportunities for surgical residents in Germany is not easily accessible for all subspecialties. It required considerable effort to gather the information presented in this manuscript, indicating that residents may struggle with finding information about available courses as well. Apart from few exceptions, no clearly defined recommendation on specific courses to attend and their suggested chronological order is presented by the professional associations. Residents and their supervisors are obliged to detect needs for additional training by themselves without a common structure and defined learning objectives they can refer to. This may change gradually with the implementation of new, competence-based WBO in 2018.

The working environment with only little space for education and research challenges additional training opportunities: out of many interesting courses offered both by professional organizations and commercial providers, only few can be attended due to limited time and financial resources. There is no information about the quality of the programs available (apart from personal recommendations by peers who have attended the courses before). Most of them do not state clearly defined learning objectives or the didactic methods applied. Therefore, it is difficult for residents as well as their training hospitals to select appropriate courses that fit the learner’s educational needs and level of experience.

Furthermore, the contents and didactic concepts of the programs are not considered in this review. Further research analyzing the training courses for their quality and effect on resident training is needed. It should be assessed if and to what extent courses and structured training programs improve surgical resident training. Ultimately, the quality of surgical training will have to be measured by the quality of patient care delivered.

The need for optimally trained surgeons persists to ensure the highest standard of safe and comprehensive surgical care. Insufficient knowledge and limited experience have been shown to entail medical errors and therefore affect patient safety [27]. Surgical residents want to deliver the highest quality of patient care and achieve the same high standards as their teachers and predecessors. To acquire and maintain procedural competency, there is a need for repetitive practice of technical skills. Studies have shown that in the surgical management of certain diseases a more favorable patient outcome is correlated with increased volume [28], [29]. A sufficient caseload and repetitive training is necessary to build expertise [30]. But with increasingly limited time available for training and spent in the OR, the need for additional training opportunities arises. Less working hours and diminishing on-the-job training mean that additional external learning opportunities should be created.

It has been shown that medical simulation is effective and that medical education in patient care settings can be complemented by simulation [31], [32]. Skills labs and training courses can enhance procedural learning, especially when it comes to high-stake procedures such as laparoscopy [33], [34]. This is especially urgent in specialties with a broad spectrum. Some procedures are rare or carry a high risk for the patient, which may entail limited opportunities to train residents. In these situations, simulation can provide the opportunity to learn new techniques and consolidate skills in a safe environment without putting patients at risk.

Ultimately, the aim of postgraduate medical education should not focus on time spent in clinical rotations or the number of medical procedures performed but on the demonstration of competence in delivering medical care to patients [35].

To enhance surgical training in Germany, there will have to be a change in surgical residency programs, and structured methods of postgraduate medical education are required. A pathway of planned learning should be laid out and designed to facilitate the progressive development of specified competencies. Training outside of teaching hospitals and structured programs should be implemented in surgical resident training in Germany.

The development and supervision of structured resident teaching programs should preferably be performed by the surgical associations rather than commercial institutes to ensure their independence of economic interests. Nonetheless, commercial providers of surgical training can play a major role in training programs (e.g. by providing high-quality simulation training courses).

The challenge – and opportunity – for the next decade will be to improve upon existing efforts in surgical specialist training and to implement quality guidelines and didactic structure in alignment with the new competence-based WBO.

## Supplementary Material

The article (iss-2018-0033) offers reviewer assessments as supplementary material.
