# Supplementary material for: Structured surgical residency training in Germany: an overview of existing training programs in 10 surgical subspecialties
Source: Innov Surg Sci. 2019 May 10;4(1):15–24. doi: 10.1515/iss-2018-0033 (PMC6754064; doi:10.1515/iss-2018-0033)
Supplement: Supplementary file 1 [file iss-4-20180033-s001.pdf]

Reviewer Assessment

Sabine Drossard*

Structured surgical residency training in Germany: an overview of existing training programs in 10 surgical subspecialties

https://doi.org/10.1515/iss-2018-0033
Received September 28, 2018; accepted April 12, 2019

***Corresponding author: Sabine Drossard,** Klinik für Kinderchirurgie und Kinderurologie, Universitätsklinikum Augsburg, Stenglinstraße 2, 86156 Augsburg, Germany, E-mail: sabine.drossard@uk-augsburg.de

Reviewers' Comments to Original Submission

Reviewer 1: Ernst Klar

Dec 16, 2018

Reviewer Recommendation Term:	Revise with Major Modifications
Overall Reviewer Manuscript Rating:	60
Is the subject area appropriate for you?	4
Does the title clearly reflect the paper's content?	2
Does the abstract clearly reflect the paper's content?	2
Do the keywords clearly reflect the paper's content?	3
Does the introduction present the problem clearly?	3
Are the results/conclusions justified?	2
How comprehensive and up-to-date is the subject matter presented?	3
How adequate is the data presentation?	4
Are units and terminology used correctly?	N/A
Is the number of cases adequate?	N/A
Are the experimental methods/clinical studies adequate?	1 - Low/No
Is the length appropriate in relation to the content?	4
Does the reader get new insights from the article?	3
Please rate the practical significance.	3
Please rate the accuracy of methods.	1 - Low/No
Please rate the statistical evaluation and quality control.	N/A
Please rate the appropriateness of the figures and tables.	4
Please rate the appropriateness of the references.	4
Please evaluate the writing style and use of language.	3
Please judge the overall scientific quality of the manuscript.	2
Are you willing to review the revision of this manuscript?	Yes

Open Access. © 2019 Drossard S., published by De Gruyter. This work is licensed under the Creative Commons Attribution 4.0 Public License.

Comments to Author:
The manuscript is laid out to provide an overview of existing courses and programs for surgical residents in Germany including the current structure of surgical training. Furthermore approaches should be developed to implement more structure in the curriculum.

Two methods were used to achieve these goals: 1) a survey amongst young surgeons, 2) a search review of websites of surgical societies. A broad description of training courses of the different societies is given. A summary is displayed in a table in a transparent fashion.

The discussion is focused on the conclusion that training should be implemented outside of teaching hospitals and structured programs in surgical resident training in Germany.

Comments:
The manuscript comprises a lot of effort represented by the poll among young surgeons and the website search and profile analysis of surgical societies. However the two aspects of the study are not represented adequately in the manuscript.

How many young surgeons were addressed in the survey, what was the percentage of feedback? Why is the questionnaire not mentioned? How many questions of what content? It is not made clear in the manuscript what conclusions could be drawn from that poll. One aim of the study was to describe the structure of surgical training in Germany. One would anticipate that a main part of the manuscript would be the result of the poll in describing which aspects of the training system are ultimately reaching the trainees. The authors criticize that the courses offered by the different scientific societies depend „on personal motivation and the generosity of the hospitals“. It would be interested to know whether this statement is supported by the poll result.

The manuscript gives the impression to mainly reflect the result of the website search by the authors while the survey is submerged.

The following contradiction should be resolved: on the one hand it is correctly analyzed that there is less and less capacity to leave hospital routine in order to attend the high quality courses outside. On the other hand the last sentence of the abstract expresses that training outside of teaching hospitals should be implemented. How should this work?

Recommendation: The authors should first gain clarity on the type of the manuscript: original paper or review? I recommend to expand the poll, combine it with what has been nicely shown here, and publish it as an original article. The current title of the work reflects a review. The conclusions should then be based on the deficits. This needs deeper evaluation especially of the survey. For instance, the training courses offered are often of very high quality. It would be important to know how many young surgeons are deprived of participation by circumstances in their specific institution.
This manuscript needs a thorough revision.

Reviewer 2: anonymous

Oct 24, 2018

**Reviewer Recommendation Term:**	Revise with Major Modifications
**Overall Reviewer Manuscript Rating:**	50
Is the subject area appropriate for you?	4
Does the title clearly reflect the paper's content?	3
Does the abstract clearly reflect the paper's content?	3
Do the keywords clearly reflect the paper's content?	2
Does the introduction present the problem clearly?	3
Are the results/conclusions justified?	3
How comprehensive and up-to-date is the subject matter presented?	3
How adequate is the data presentation?	1 - Low/No
Are units and terminology used correctly?	N/A
Is the number of cases adequate?	N/A
Are the experimental methods/clinical studies adequate?	N/A
Is the length appropriate in relation to the content?	3
Does the reader get new insights from the article?	2
Please rate the practical significance.	3
Please rate the accuracy of methods.	1 - Low/No
Please rate the statistical evaluation and quality control.	N/A
Please rate the appropriateness of the figures and tables.	3

Please rate the appropriateness of the references.	2
Please evaluate the writing style and use of language.	3
Please judge the overall scientific quality of the manuscript.	2
Are you willing to review the revision of this manuscript?	Yes

Comments to Author:
The authors are addressing an interesting field. They present a description on surgical residency training in Germany complaining deficits with regards on structures and contents. They mainly focus on surgical skills courses offered by 8 surgical associations and 2 commercial institutions. Results are obtained by “asking” members of the German young surgeons association “about the existence of structured residence training” and “personal information” and by “online research”. As the authors stated, information remains incomplete.
Virtual reality is only shortly mentioned in the discussion. To increase the value of the manuscript, data from reports on the effects of surgical training on models, animals or simulators for improving surgical education should be added.

Authors’ Response to Reviewer Comments

Jan 28, 2019

Dear Reviewers,

thank you very much for your thorough review, the helpful comments and constructive suggestions. I adapted the manuscript accordingly and feel that it gained in quality and clarity.
Please find a detailed response to your suggestions below.

*Introduction. Listing the surgical sub-specialities, neurosurgery was missed out. Please stress that the BÄK is proposing, Landesärztekammern are responsible for/ and implementing WBO.

The difference BÄK/Landesärztekammern was inserted.

The listing of surgical specialites and this article originally focussed on the subspecialties of surgery that are part of the „common trunk“. Therefore only 8 subspecialties are listed. Neurosurgery (and Oral/Maxillofacial Surgery) was deliberately left out because it is not part of the common trunk.
Additional research was carried out and the test was expanded, so that now all 10 subspecialties that are represented in Deutsche Gesellschaft für Chirurgie are covered in this manuscript.

*2. Material & Methods. About the “members asked” How many persons have been asked? Please specify whether it was an unstructured talk, or a structured questioning with a focussed question?
3. Please specify the process of your online search, i.e. were just the websites if the surgical associations used?

This paragraph was expanded and specified.

*Reviewer #1: The manuscript is laid out to provide an overview of existing courses and programs for surgical residents in Germany including the current structure of surgical training. Furthermore approaches should be developed to implement more structure in the curriculum. Two methods were used to achieve these goals: 1) a survey amongst young surgeons, 2) a search review of websites of surgical societies. A broad description of training courses of the different societies is given. A summary is displayed in a table in a transparent fashion. The discussion is focused on the conclusion that training should be implemented outside of teaching hospitals and structured programs in surgical resident training in Germany.

Comments:
The manuscript comprises a lot of effort represented by the poll among young surgeons and the website search and profile analysis of surgical societies. However the two aspects of the study are not represented adequately in the manuscript.
How many young surgeons were addressed in the survey, what was the percentage of feedback? Why is the questionnaire not mentioned? How many questions of what content? It is not made clear in the manuscript what conclusions could be drawn from that poll. One aim of the study was to describe the structure of surgical training in Germany. One would anticipate that a main part of the manuscript would be the result of the poll in describing which aspects of the training system are ultimately reaching the trainees. The authors criticize that the courses offered by the different scientific societies depend “on personal motivation and the generosity of the hospitals”. It would be interested to know whether this statement is supported by the poll result.
The manuscript gives the impression to mainly reflect the result of the website search by the authors while the survey is submerged.

The Materials and Methods paragraph was expanded and specified. Please excuse the confusion about this part of the manuscript.
In fact no survey was carried out. The main method was a web-search. The information gathered was checked against and complemented by personal information from representatives of the surgical associations (who are listed individually in the acknowledgements).

The survey mentioned in the results was carried out by BDC in 2015, this part was moved to the discussion, as it is of course not a result of this research and was therefore incorrectly placed in the results paragraph, leading to confusion. It is now clearly stated that the mentioned survey was carried out by BDC.

This article does not claim to completely list every training course available today. It acts as a first comprehensive overview of different approaches to structured surgical training in Germany that are currently in use. As no other overview is available at the moment the author believes the study is of great interest, however it can only be seen as a starting point for further research.

*The following contradiction should be resolved: on the one hand it is correctly analyzed that there is less and less capacity to leave hospital routine in order to attend the high quality courses outside. On the other hand the last sentence of the abstract expresses that training outside of teaching hospitals should be implemented. How should this work?

Thank you for this suggestion. Without question this is an important and conflictual aspect that was only briefly discussed in the manuscript. Actually the author states that because there are less learning opportunities inside the hospital additional external courses should be considered. The discussion has been expanded and concretised to reflect this aspect.

*Recommendation: The authors should first gain clarity on the type of the manuscript: original paper or review? I recommend to expand the poll, combine it with what has been nicely shown here, and publish it as an original article. The current title of the work reflects a review. The conclusions should then be based on the deficits. This needs deeper evaluation especially of the survey. For instance, the training courses offered are often of very high quality. It would be important to know how many young surgeons are deprived of participation by circumstances in their specific institution.
This manuscript needs a thorough revision.

Thank you again for your comments. The manuscript aims to function as a first review of available surgical training programs in Germany, as there is no such outline available at present. It focuses on listing and describing the structure of existing programs. Their content and educational concepts were not examined. This was concretised in the manuscript.

Further research analysing the training courses for their quality and effect on resident training is needed.
The subsequent question is, as you have pointed out, how the training affects surgical residents and ultimately how patient care is influenced by it. This of course is a complex issue where further research is needed.

How many young surgeons are deprived of participation can only be estimated, but the quoted survey of BDC implies that 95% of resident were granted up to 5 days of leave for external trainings. Further research is indicated to identify the amount of residents who are facing difficulties at their institution when requesting to attend external training programs. Hopefully institutions like BDC and PFJC will be able to collect more data in future surveys.

*Reviewer #2: The authors are addressing an interesting field. They present a description on surgical residency training in Germany complaining deficits with regards on structures and contents. They mainly focus on surgical skills courses offered by 8 surgical associations and 2 commercial institutions. Results are obtained by "asking" members of the German young surgeons association "about the existence of structured residence training" and "personal information" and by "online research". As the authors stated, information remains incomplete.

Thank you for your constructive comments on the manuscript.
Information remains incomplete but was extended to include data on Neurosurgery and Oral/Maxillofacial Surgery.

*Virtual reality is only shortly mentioned in the discussion. To increase the value of the manuscript, data from reports on the effects of surgical training on models, animals or simulators for improving surgical education should be added.

Indeed virtual reality training is not elaborated on, as the research showed that this training method is not commonly used for surgical resident training at the moment. Most programs described consist mainly of lectures and seminars, supplemented by some fraction of practical skills training.

Data supporting the effectiveness of simulation for surgical training was added to support the request of structured training and practical courses. The implementation of the new comptence-based MWBO and the prospect of changes to surgical education was added.
Thank you again for your suggestions. I feel that the focus of the discussion was improved considerably.

Reviewers' Comments to Revision

Reviewer 1: Ernst Klar

Apr 01, 2019

Reviewer Recommendation Term:	Accept with Minor Revision
Overall Reviewer Manuscript Rating:	75
Is the subject area appropriate for you?	4
Does the title clearly reflect the paper's content?	3
Does the abstract clearly reflect the paper's content?	4
Do the keywords clearly reflect the paper's content?	4
Does the introduction present the problem clearly?	4
Are the results/conclusions justified?	4
How comprehensive and up-to-date is the subject matter presented?	4
How adequate is the data presentation?	3
Are units and terminology used correctly?	4
Is the number of cases adequate?	N/A
Are the experimental methods/clinical studies adequate?	N/A
Is the length appropriate in relation to the content?	4
Does the reader get new insights from the article?	4
Please rate the practical significance.	4
Please rate the accuracy of methods.	4
Please rate the statistical evaluation and quality control.	N/A
Please rate the appropriateness of the figures and tables.	4
Please rate the appropriateness of the references.	4
Please evaluate the writing style and use of language.	4
Please judge the overall scientific quality of the manuscript.	3
Are you willing to review the revision of this manuscript?	No: No re-review necessary

Comments to Author:

The authors have considerably added structure to the manuscript and have made interpretation clearer.
The abstract remains to be modified currently being all narrative. Some parameters should be included:
- How many societies were included?
- The characteristics of the courses should be mentioned according to the horizontal bar of Table 1: e.g. „The courses were analyzed according to seven characteristics….”.
- In „Results” the respective number of courses should be added instead of „...several…” or „…some…”
In summary the manuscript has been much improved and is now limited by the study design, which is stated clearly by the authors.
Realizing these obvious limitations which cannot be improved the manuscript can now be published.
Minor:
“The quantity and variety of courses offered varies considerable…”
Correct: „…vary considerably…“
(pages are not numbered!)

Reviewer 2: anonymous

Feb 13, 2019

**Reviewer Recommendation Term:**	Reject
**Overall Reviewer Manuscript Rating:**	30
Is the subject area appropriate for you?	4
Does the title clearly reflect the paper’s content?	4
Does the abstract clearly reflect the paper’s content?	3
Do the keywords clearly reflect the paper’s content?	3
Does the introduction present the problem clearly?	4
Are the results/conclusions justified?	2
How comprehensive and up-to-date is the subject matter presented?	3
How adequate is the data presentation?	N/A
Are units and terminology used correctly?	N/A
Is the number of cases adequate?	N/A
Are the experimental methods/clinical studies adequate?	N/A
Is the length appropriate in relation to the content?	3
Does the reader get new insights from the article?	2
Please rate the practical significance.	2
Please rate the accuracy of methods.	1 - Low/No
Please rate the statistical evaluation and quality control.	N/A
Please rate the appropriateness of the figures and tables.	2
Please rate the appropriateness of the references.	3
Please evaluate the writing style and use of language.	3
Please judge the overall scientific quality of the manuscript.	1 - Low/No
Are you willing to review the revision of this manuscript?	Yes

Comments to Author:

Thank you for working on your manuscript which has improved in quality. It includes a presentation of answers, received from “open questions” from different surgical specialists and the results from the search for surgical skills training programs. The methods used are now more precisely indicated. Results are describing some courses for surgical training skills courses and the problems with postgraduate education in Germany. At the end the authors complain of the insufficient situation in Germany and require the implementation of quality guidelines and competence based specialty training regulations. It would be interesting to know more about the authors’ conception.
Two further remarks.
1. In paragraph Surgical training in Germany (row 5) is written: “. . .any department of the 8 mentioned subspecialties . . .” This is not completely correct. Surgical education is recognized only by authorized surgeons (Weiterbildungsermächtigte).
2. Discussion, 2nd section (row 1-2): Adding “literature” to the sentence “. . . available on the internet and personal statements” is recommended.

Editor's Comment to Author

Apr 1, 2019
Dear Author,

thank you for submitting your revised manuscript that has greatly improved from the first version.
We have received two reviews, one suggesting to accept the manuscript without further review after minor revisions, the other to revise and resubmit/reject, due to the "incompleteness of the manuscript" and due to the lack of international comparison. However, this article will be part of a special issue on the situation of Young Surgeons that has an article included and already accepted comparing different international education systems to the German system. This apparent "incompleteness" is thus redundant through another article and does not need to be a part of this article. The other shortcoming remarked by reviewer 2 is inherent to the type of article and analysis and as fittingly remarked by reviewer 1 it is sufficiently discussed during the limitations and discussion session.

In summary, understanding the critique of reviewer 2, I can completely follow the advice of reviewer 1 In that this article can be published upon completing the pending minor revisions. It should be labelled as an opinion paper, though.

Please refer to the minor points by the reviewers from below.

Thank you for contributing to this special issue.

Authors' Response to Reviewer Comments

Apr 11, 2019
Dear Reviewers,
thank you for your repeated review, the positive feedback on my manuscript and your further suggestions.
I adapted the manuscript again according to your suggestions.
Please find a detailed response below.
Reviewer #1: The authors have considerably added structure to the manuscript and have made interpretation clearer.
The abstract remains to be modified currently being all narrative. Some parameters should be included:
- How many societies were included?
- The characteristics of the courses should be mentioned according to the horizontal bar of Table 1: e.g. „The courses were analyzed according to seven characteristics….".
In „Results" the respective number of courses should be added instead of „…several…" or „…some…"

The abstract was re-written to include more information and better reflect the content of the manuscript.

In summary the manuscript has been much improved and is now limited by the study design, which is stated clearly by the authors. Realizing these obvious limitations which cannot be improved the manuscript can now be published.
Minor:
"The quantity and variety of courses offered varies considerable…"
Correct: "…vary considerably…"
(pages are not numbered!)

Grammar was corrected.
(A numbering of pages was not possible when submitting text via the EditorialManager.)

Reviewer #2: Thank you for working on your manuscript which has improved in quality. It includes a presentation of answers, received from "open questions" from different surgical specialists and the results from the search for surgical skills training programs. The methods used are now more precisely indicated. Results are describing some courses for surgical training skills courses and the problems with postgraduate

education in Germany. At the end the authors complain of the insufficient situation in Germany and require the implementation of quality guidelines and competence based specialty training regulations. It would be interesting to know more about the authors' conception.
Two further remarks.
In paragraph Surgical training in Germany (row 5) is written: "...any department of the 8 mentioned subspecialties ..." This is not completely correct. Surgical education is recognized only by authorized surgeons (Weiterbildungsermächtigte).

Thank you for noting this detail, it was corrected in the text.

2. Discussion, 2nd section (row 1-2): Adding "literature" to the sentence ". . . available on the internet and personal statements" is recommended.

This was added as proposed.
